# Supplementary material for: The Effects of Different Feeding Regimes on Body Composition, Gut Microbial Population, and Susceptibility to Pathogenic Infection in Largemouth Bass
Source: Microorganisms. 2023 May 22;11(5):1356. doi: 10.3390/microorganisms11051356 (PMC10221583; doi:10.3390/microorganisms11051356)
Supplement: Supplementary file 1 [file microorganisms-11-01356-s001.zip › microorganisms-2363910-supplementary.pdf]

microorganisms

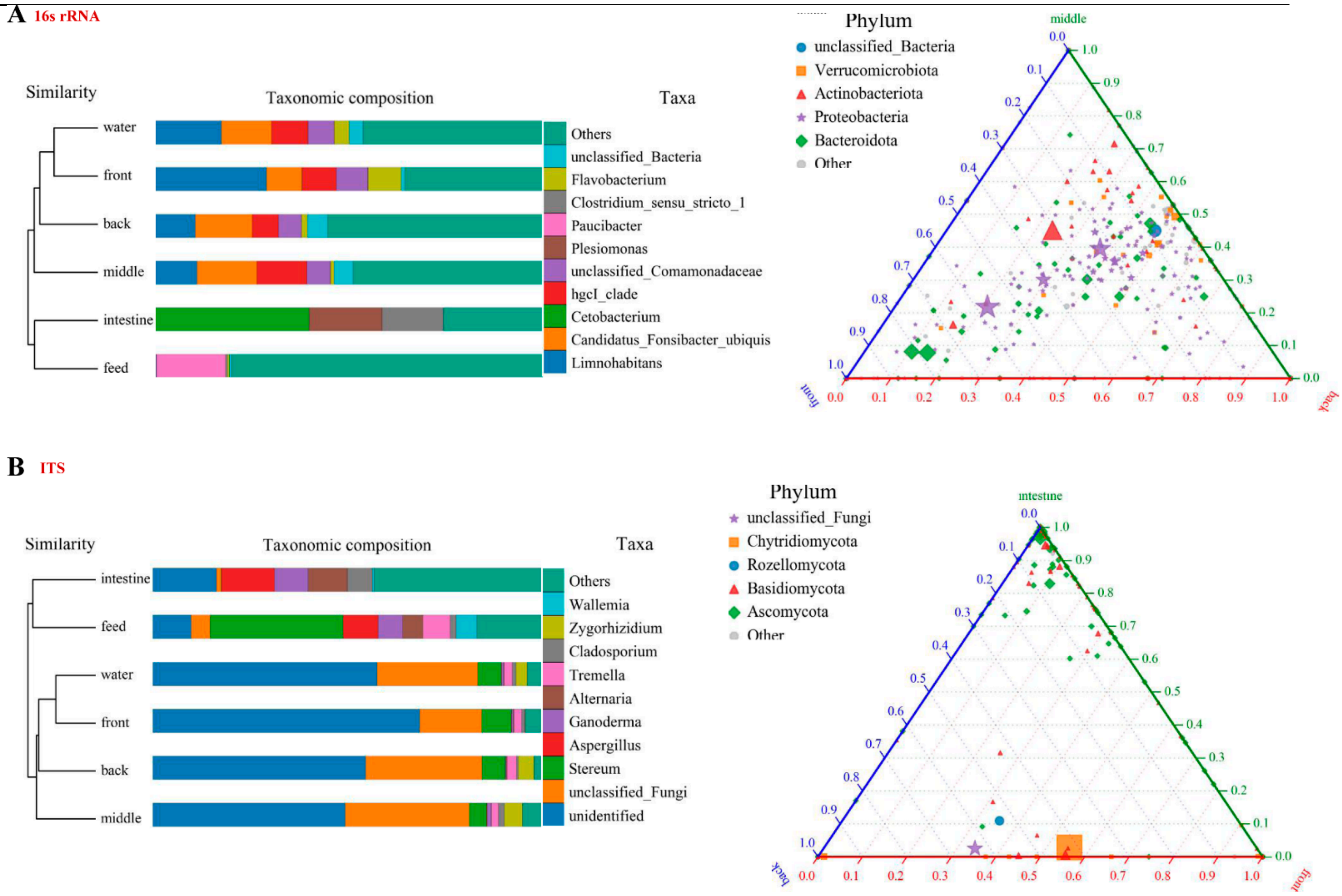

Figure S1. The combined UPGMA clustering tree and histogram of species composition. Taxonomic categories A was the results for 16S rRNA, while B for ITS. This diagram. Sample clustering trees on the left were constructed based on four distance algorithms, which shown the similarity between samples. Species composition in genus level were shown as histogram placed on the right in order to visualize similarity in species abundance. Ternary diagram uses an equilateral triangle to describe the ratio relationship of different attributes of three variables. Three samples have their own colors, dots with different colors represent the species of the corresponding samples. Three edges are used to measure the species richness of samples in corresponding colors. Circles in the diagram represent species classification of all genus level (Other levels can be selected) contained in a phylum level, size of the circle represents the average relative richness of species. Color circles in example diagram represents the species classification of phylum level (Other levels can be selected) with top 5 abundance.

microorganisms MDPI

Table S1 Growth performance, feed component and α/β-diversity of sequencing in LB[#]

	Item	PF (*n*=7)	WF (*n*=8)	PI (*n*=7)	PFI (*n*=8)	Commercial feed (dry weight)	Iced fish (dry weight)
	WG	262.38 ± 12.34	231.42 ± 17.46	252.77 ± 21.12	280.66 ± 9.48		
	SGR	2.86 ± 0.15	3.02 ± 0.21	2.94 ± 0.30	2.77 ± 0.17		
	CF	2.57 ± 0.05	2.64 ± 0.07	2.49 ± 0.08	2.68 ± 0.04		
	FI	13.57 ± 0.57	13.65 ± 0.44	14.71 ± 0.39	13.96 ± 0.13	/	/
	FER	0.94 ± 0.08	0.89 ± 0.09	0.73 ± 0.09	0.76 ± 0.04		
	SR	95.16 ± 5.11	90.45 ± 4.09	92.62 ± 3.27	97.74 ± 6.65		
	Moisture	30.50 ± 4.92	30.32 ± 5.77	48.46 ± 6.81	30.18 ± 9.11	12.50 ± 0.11	48.47 ± 0.39*
	Crude protein	46.85 ± 0.32	46.41 ± 0.45	23.18 ± 0.61	44.92 ± 0.37	45.60 ± 1.04	13.19 ± 0.11*
	Crude lipid	5.92 ± 1.00	5.70 ± 0.82	4.73 ± 0.35	5.47 ± 0.72	4.90 ± 0.17	5.44 ± 0.25
	Crude ash	2.73 ± 0.14	2.63 ± 0.22	1.47 ± 0.30	1.77 ± 0.27	16.40 ± 0.33	6.72 ± 0.26*
	Crude fiber	3.81 ± 0.09	3.73 ± 0.07	0.54 ± 0.10	3.61 ± 0.08		
	Nitrogen-free extract	40.28 ± 7.23	41.64 ± 5.89	45.35 ± 4.88	42.32 ± 6.21		
	Energy (MJ/kg)	19.71 ± 0.21	19.68 ± 0.34	21.44 ± 0.46	20.78 ± 0.39		
OTUs (16s/ITS)	Commercial feed (*n*=3)	190 ± 17/123 ± 17					
	Iced fish (*n*=3)	236 ± 21/66 ± 7					
	Water (*n*=3)	129 ± 16/103 ± 12	164 ± 23/ 99 ± 5	155 ±13/108 ± 12	133 ± 13/146 ± 13		
	intestine	172 ± 55/95 ± 28	160 ± 54/82 ± 9	167 ± 7/85 ± 4	153 ± 20/71 ± 3		

Ace (16s/ITS)	Commercial feed (*n*=3)	194.08 ± 13.61/126.58 ± 19.81			
	Iced fish (*n*=3)	249.10 ± 20.25/66.33 ± 4.67			
	Water (*n*=3)	180.93 ±13.15/124.91 ± 15.26	219.71 ± 12.05/118.80 ± 14.91	200.82 ± 13.33/109.13 ± 29.33	165.30 ± 8.00/148.46 ± 13.26
	intestine	230.31 ± 62.41/95.36 ± 6.74	203.90 ± 76.56/82.01 ± 2.84	218.22 ± 5.06/85.55 ± 4.25	217.14 ± 54.71/67.32 ± 4.83
Chao1 (16s/ITS)	Commercial feed (*n*=3)	194.91 ± 14.22/129.06 ± 21.73			
	Iced fish (*n*=3)	254.37 ± 23.75/66.33 ± 4.67			
	Water (*n*=3)	166.76 ± 15.63/116.45 ± 5.89	216.24 ± 13.21/120.83 ± 4.89	194.71 ± 27.33/109.94 ± 13.07	168.46 ±10.82/148.33 ± 12.13
	intestine	218.22 ± 63.88/95.25 ± 6.13	210.87 ± 77.53/81.71 ± 2.87	217.17 ± 11.85/84.97 ± 4.25	199.55 ± 33.17/71.75 ± 4.37
Simpson (16s/ITS)	Commercial feed (*n*=3)	0.02 ± 0.01/0.09 ± 0.01			
	Iced fish (*n*=3)	0.16 ± 0.01/0.39 ± 0.02			
	Water (*n*=3)	0.16 ± 0.01/0.22 ± 0.04	0.10 ± 0.02/0.21 ± 0.04	0.13 ± 0.01/0.11 ±0.01	0.09 ± 0.01/0.11 ±0.01
	intestine	0.09 ± 0.05/0.10 ± 0.02	0.12 ± 0.11/0.20 ± 0.01	0.19 ± 0.09/0.18 ± 0.02	0.21 ± 0.16/0.20 ± 0.02
Shannon (16s/ITS)	Commercial feed (*n*=3)	4.51 ± 0.33/3.14 ± 0.04			
	Iced fish (*n*=3)	3.63 ± 0.09/1.99 ± 0.11			
	Water (*n*=3)	2.89 ± 0.11/2.29 ± 0.05	3.45 ± 0.05/2.27 ± 0.04	3.22 ± 0.82/3.00 ± 0.08	3.22 ± 0.41/3.26 ± 0.14
	intestine	3.29 ± 0.47/3.40 ± 0.41	3.27 ± 0.69/2.88 ± 0.28	2.78 ± 0.38/3.21 ± 0.13	2.81 ± 0.71/2.79 ± 0.13

[#]Statistically significant effects are shown with the star (*). The α-diversity was revealed among different groups. During the α-diversity analysis, the bacterial community indices included Chao1, Ace, Shannon and Simpson.

Table S2 The water quality indexes and production performance among different treatment groups#

Groups	Water quality indexes from June 26th to July 2th 2017									Product yield (kg/m^2)	Disease time/year	Death rate(%)	Fatty liver disease rate(%)	Fat ratio(%)
	T(°C)	pH	Salinity(‰)	DO (mg/L)	SD(cm)	TSS(mg/L)	TN(mg/L)	TP(mg/L)	COD_{Mn}(mg/L)					
PF(*n* = 7)	27.5±0.2	7.8±0.1	1.0±0.0	4.8±0.1^{b}	30±1^{a}	26±2^{a}	5.02±0.11^{a}	0.35±0.01^{a}	24.6±0.1	1.20-1.50^{b}	1	6.5±0.7^{bc}	16±2^{b}	4.80±0.04^{b}
WF(*n* = 8)	28.0±0.1	7.8±0.1	1.0±0.0	5.6±0.2^{a}	24±1^{b}	18±1^{b}	4.68±0.08^{b}	0.23±0.01^{b}	25.0±0.2	68.18^{a}	1	5.2±0.6^{c}	5±0^{c}	1.79±0.02^{c}
PI(*n* = 7)	27.5±0.1	7.6±0.1	1.0±0.0	4.5±0.1^{b}	36±2^{a}	29±2^{a}	5.92±0.09^{a}	0.37±0.02^{a}	26.6±0.1	1.27-1.65^{b}	2	10.5±0.9^{a}	28±2^{a}	6.80±0.05^{a}
PFI(*n* = 8)	27.5±0.1	7.7±0.1	1.0±0.0	4.7±0.1^{b}	32±2^{a}	28±2^{a}	5.61±0.14^{a}	0.36±0.01^{a}	26.6±0.1	1.20-1.50^{b}	2	8.0±0.7^{b}	20±1^{b}	5.20±0.06^{ab}

#PF pond used whole-course commercial feed, WF water tank used whole-course commercial feed, PI pond used whole-course iced fish, PFI pond used the commercial feed (8% feeding ratio for 5 months) and iced fish (8% feeding ratio for 6 months). One-way ANOVA was used to test for differences among treatments, and data standed for significant differences with the lowercase letters.
